# Breeding for improved digestibility and processing of lignocellulosic biomass in *Zea mays*

**DOI:** 10.3389/fpls.2024.1419796

**Published:** 2024-07-26

**Authors:** Yasmine Vanhevel, Astrid De Moor, Hilde Muylle, Ruben Vanholme, Wout Boerjan

**Affiliations:** ^1^ Department of Plant Biotechnology and Bioinformatics, Ghent University, Ghent, Belgium; ^2^ Center for Plant Systems Biology, VIB, Ghent, Belgium; ^3^ Plant Sciences Unit, Institute for Agricultural and Fisheries Research, Melle, Belgium

**Keywords:** maize, lignin, breeding, lignin engineering, digestibility, saccharification

## Abstract

Forage maize is a versatile crop extensively utilized for animal nutrition in agriculture and holds promise as a valuable resource for the production of fermentable sugars in the biorefinery sector. Within this context, the carbohydrate fraction of the lignocellulosic biomass undergoes deconstruction during ruminal digestion and the saccharification process. However, the cell wall’s natural resistance towards enzymatic degradation poses a significant challenge during both processes. This so-called biomass recalcitrance is primarily attributed to the presence of lignin and ferulates in the cell walls. Consequently, maize varieties with a reduced lignin or ferulate content or an altered lignin composition can have important beneficial effects on cell wall digestibility. Considerable efforts in genetic improvement have been dedicated towards enhancing cell wall digestibility, benefiting agriculture, the biorefinery sector and the environment. In part I of this paper, we review conventional and advanced breeding methods used in the genetic improvement of maize germplasm. In part II, we zoom in on maize mutants with altered lignin for improved digestibility and biomass processing.

## Introduction

Maize, also known as corn, plays a multifaceted role in agriculture, serving as a crucial resource for food, feed and the production of basic chemicals such as fuels, plastics, etc. Maize silage represents the areal part of maize – including leaves, stems, cobs and seeds – stored under anaerobic conditions for conservation, to be used as ruminant feed (Barrière, 2017). The primary energy source in maize silage comes from starch in the kernels and from the cell wall carbohydrates. The cell wall, in the animal feed field known as neutral detergent fiber (NDF), is composed mainly of two types of carbohydrates, cellulose and hemicellulose, and the aromatic heteropolymer, lignin. NDF digestibility is influenced by the genetic background, the environmental conditions, field management and timing of harvest, and typically varies between 40 to 50% (Allen et al., 2003; Barrière, 2017). At the cell wall level, NDF digestibility is primary influenced by the lignin content, lignin composition and the ferulate cross-linkages between lignin and hemicelluloses (Wolf et al., 1993; Fontaine et al., 2003; Grabber et al., 2009; Courtial et al., 2013; Barrière, 2017). Consequently, breeding efforts to improve the NDF digestibility often target the lignin characteristics^1^.

Besides its use as ruminant feed, maize lignocellulosic biomass (i.e., maize stover consisting of the leaves, stems and cobs without seeds) has been identified as a promising resource for the biorefinery (Torney et al., 2007; Vermerris et al., 2007; Lorenzana et al., 2010; van der Weijde et al., 2013; Torres et al., 2015b; Saratale et al., 2019). During biorefining, the carbohydrate fraction of the lignocellulosic biomass is enzymatically deconstructed into primary sugars, a process called saccharification. These primary sugars can then be used in fermentation reactions to produce renewable materials and biofuels (Lips, 2022). Similar to ruminal digestibility, the intrinsic resistance of the maize stover to enzymatic degradation, also known as biomass recalcitrance, is mainly caused by the presence of lignin (Torres et al., 2015a). Therefore, breeding towards a reduced lignin amount, an altered lignin composition, or an altered interaction between lignin and hemicelluloses can be advantageous to improve both feed digestibility and the industrial saccharification process (Torres et al., 2016). In this review, we start by providing a brief history of conventional maize breeding methods, followed by an overview of more advanced breeding strategies. Next, we focus on maize mutants and transgenic lines with a modified lignin content and composition, and their effect on digestibility and biomass processing efficiency.

## Part I. The past, present and future of maize breeding

### The origin of *Zea mays*

Maize (*Zea mays L*. spp. *mays*) is currently one of the most important staple crops, along with rice and wheat, worldwide (FAO, 2023). The origin of maize has been studied extensively; phylogenetic analyses and archaeological research show a direct ancestral link to two wild grass subspecies commonly known as teosinte (*Z. mays* ssp. *parviglumis* and *Z. mays* ssp. *mexicana*) (Yang et al., 2023). Maize domestication started 7.000 to 10.000 years ago in the tropical lowlands of present-day Mexico when indigenous Americans discovered its potential as food. From its origin, maize spread northwards and southwards throughout the American continent, giving rise to Northern Flint and Southern Dent lineages (see later) (Tenaillon and Charcosset, 2011). This spread caused maize to diversify under genetic drift and selection, resulting in varieties adapted to different climates and soil types, from sea level to the high altitudes in the Andean mountains (Vigouroux et al., 2008; Bouchet et al., 2013; Andorf et al., 2019). In the 15^th^ and 16^th^ century, maize was introduced into Europe, Africa and Asia via European explorers and traders (Barrière et al., 2006). The early maize breeders on each of the continents played a pivotal role in the domestication process and the development of maize cultivars as we know them today. Recent advances in biotechnology and genomics provide new tools for maize breeders to further improve their cultivars and speed up the breeding process (Singh et al., 2021b).

### Modern maize breeding

In the 19^th^ century, farmers selected the best ears from the most productive and healthy plants. Their seeds were then sown in the next growth season and the selection process was repeated. This so-called *mass selection* is the oldest form of maize breeding. This method is simple and effective for fixing traits with high heritability, but not very effective for traits with low heritability, such as yield (Choo and Kannenberg, 1981; Hallauer et al., 2010). Hopkins introduced the *ear-to-row selection* method to speed up maize breeding (Hopkins, 1899). In this method, a number of maize plants with desirable phenotypes are identified and their seeds harvested separately. About 50 seeds from a single ear are then grown in a single progeny row, and allowed to open-pollinate. From these progeny rows, again the best plants are identified and allowed to open-pollinate. This process is repeated for three to six generations until the individual plants from this open-pollinated variety (OPV) start showing similarity for the desired trait (Awata et al., 2019). In the late 19^th^ century, *hybridization* became a game-changing method in maize breeding. It was noticed that the offspring (i.e., hybrids) of two different open-pollinated maize cultivars had up to 53% higher yield as compared to either parent (Beal, 1878). This phenomenon, called “heterosis” or “hybrid vigor”, has been extensively exploited in breeding programs even though the molecular basis is still poorly understood (Labroo et al., 2021; Yu et al., 2021). Breeders have primarily relied on ‘heterotic groups’ to select parents to make hybrid combinations. A heterotic group is a collection of germplasm, that, when crossed with germplasm from another heterotic group, tends to exhibit a higher degree of heterosis (on the average) than when crossed with a member of its own group (Lee, 1995). The genetic diversity of the germplasm within one heterotic group is too small to give the desired hybrid vigor effect (Akinwale, 2021). Early 20^th^ century, Shull and East independently discovered that both heterozygous and homozygous loci were present in OPVs and that fully homozygous lines can be obtained after five to seven generations of self-pollination (East, 1908; Shull, 1908, 1909). These inbred lines were often weak due to inbreeding depression, but vigor could be restored in the hybrid offspring of two different inbred lines. By continued improvement of the inbred lines, the production of ‘hybrid maize’ became a reality and hundreds of inbred lines were developed for the production of hybrids (Bennetzen and Hake, 2009; Hallauer et al., 2010). Therefore, the modern hybrid maize breeders have two main activities. First, genetically improving inbred lines by recombination and introgression of interesting alleles and, secondly, testing the combining ability between inbred lines to produce outstanding hybrids. Subsequently, these hybrids are extensively evaluated for their agronomic performance before commercialization (Lee and Tollenaar, 2007).

### Inbred line development

Inbred lines can be made in various ways, e.g., via *pedigree breeding*, *backcross breeding* or *doubled haploid breeding*. *Pedigree breeding* is a method to gradually improve a population by concentrating desirable alleles through a selection process of the best hybrids (Beckett et al., 2019; Singh et al., 2021a). In short, parents with desirable traits are crossed to generate hybrid seedstocks (in case the parental lines are heterozygous, their offspring will be highly heterogeneous). The resulting hybrids are then evaluated and the best-performing plants are self-pollinated for several generations to create inbreds. In each generation, plants with obvious defects are removed and the promising inbred lines are crossed with tester lines from different heterotic groups to evaluate their general combining ability. The newly generated inbreds are retained and the cycle is repeated until the inbreds are ready for use in cultivar development (Lee and Tollenaar, 2007).

**Figure 1:**
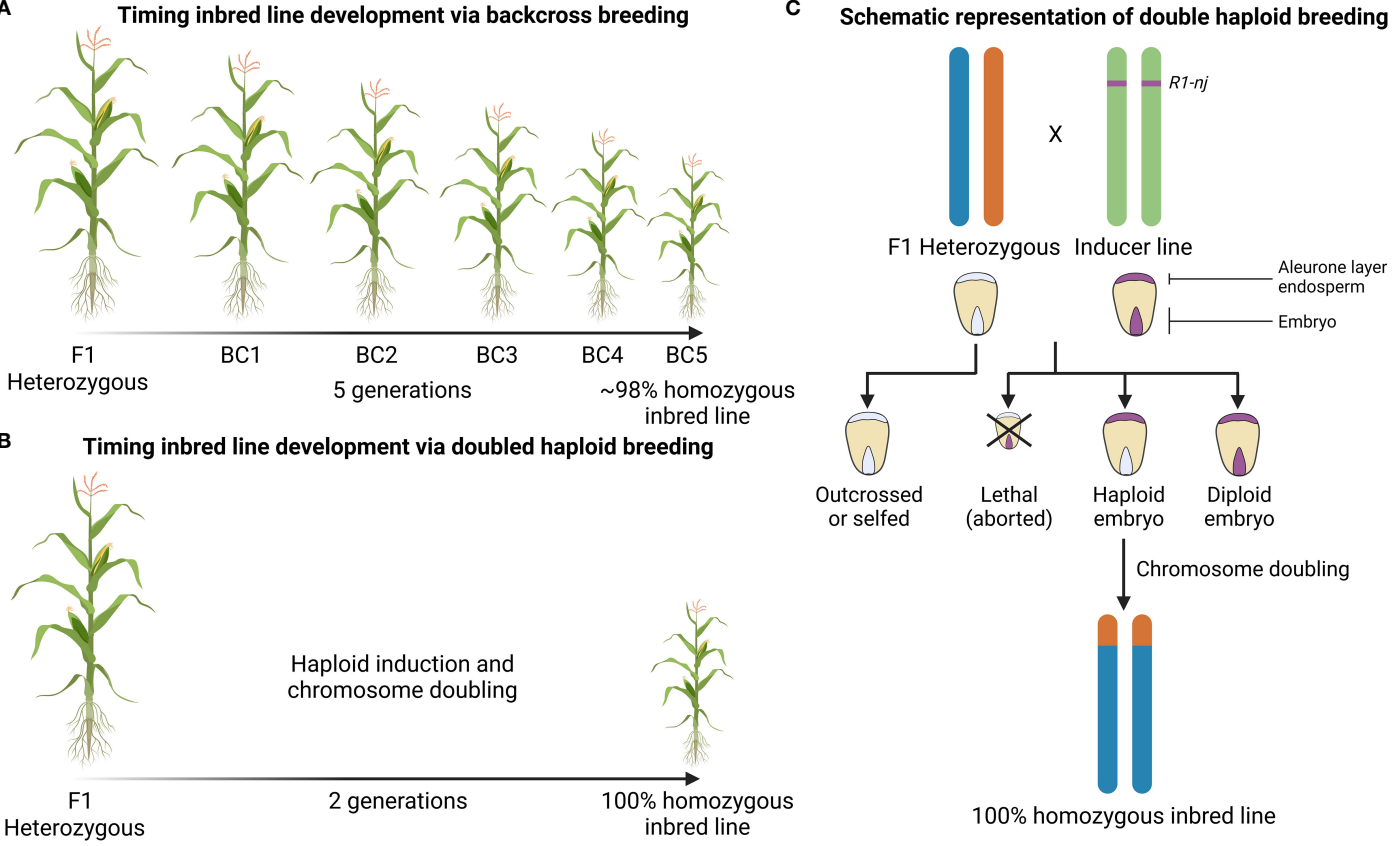
Graphical representation of inbred line development via conventional backcross breeding or doubled haploid technology. **(A)** Number of generations necessary to obtain a homozygous inbred line using conventional backcross breeding and **(B)** using doubled haploid breeding. **(C)** Identification of haploid seeds using the R1-nj color marker. The colored bars represent chromosomes. The inducer line carries the R1-nj dominant marker gene; the location on the green chromosomes does not reflect the actual physical location, but signifies its presence in the inducer line’s genome. Chromosome doubling, either artificial or spontaneous, after haploid induction is essential to obtain fertile homozygous inbred lines. The homozygous inbred line shown is just one of many possibilities, derived from a single recombinant gamete from the hybrid parent.

*Backcross breeding* allows the breeder to transfer a desired trait obtained from a donor parent into a favored elite background (also called recurrent parent). Here, the objective is to genetically recover the recurrent parent except for the desired trait. Theoretically, in each backcross (BC) generation, about 50% of the recurrent parent genome is recovered (F1 – 50%, BC1 – 75%, BC2 – 87.5%, BC3 – 93.7%, BC4 – 96.9%, BC5 – 98.4%). Donor genes can reside for example in exotic germplasm, transgenic genotypes or mutants. Typically, breeders try to recover at least 98% of the recurrent parent genome (Vogel, 2009). This process is generally slow but molecular markers can be used to reduce the number of BC generations (**Figure 1A**). Marker technology can be carried out at the seedling stage and allows to characterize multiple loci at once and minimize linkage drag, thus speeding up the breeding process (Hasan et al., 2021).

*Doubled haploid breeding* is a method to obtain pure inbred lines within a single generation (Ren et al., 2017) (**Figure 1B**). Typically, the production of stable and uniform hybrids relies on inbred line development and the latter takes multiple generations of self-crossing or back-crossing as described above. This labor intensive and time-consuming process can be overcome by the use of a genetic trick. Haploids can be generated in different ways (tissue culture from mega- and microsporocytes, natural generation by androgenesis, CENH3-mediated haploid induction and via haploid inducer lines) but making use of inducer lines is the most popular method (Ren et al., 2017; Maqbool et al., 2020; Meng et al., 2021). Coe discovered that one of his maize stocks, “stock 6”, produced 2 to 3% haploid plants when used as a male parent in crosses (Coe, 1959). As Coe’s “stock 6” was able to produce haploid progeny, it was named a “haploid inducer (HI)”. The process of haploid induction is also known as gynogenesis and is an asexual way of reproduction in which the male gamete triggers the development of an unfertilized egg into a haploid embryo (Gilles et al., 2017). In order to ease haploid identification, the HI is combined with the *R1-navajo* (*R1-nj*) gene, a dominant marker that results in a purple anthocyanin color in the kernel. This gene is expressed in the aleurone layer of the endosperm and in the embryo and allows to discriminate haploid seeds from diploid seeds (Rádi et al., 2020). The haploids will have no purple color in the embryo but will show a coloration in the aleurone layer (**Figure 1C**) (Bennetzen and Hake, 2009). Haploids are usually highly sterile and cannot undergo meiosis, but will produce pure and fertile diploid inbreds or doubled haploids after spontaneous or artificial chromosome doubling (Jackson, 2017).

The underlying major causal gene for the haploid induction in “stock 6” has been independently identified by three different research groups (Gilles et al., 2017; Kelliher et al., 2017; Liu et al., 2017). The mutation was mapped to a patatin-like phospholipase gene expressed in mature pollen and the pollen tube. This same gene was named by the three groups *NOT LIKE DAD* (*NLD*), *MATRILINEAL* (*MTL*), and *ZmPHOSPHOLIPASE A1* (*ZmPLA1*). Recently, additional mutations have been identified that are able to further boost the haploid induction rate of *NLD*/*MTL*/*ZmPLA1* mutants (Zhong et al., 2019; Li et al., 2021; Jiang et al., 2022). Modern inducer lines have 7 to 16% haploid progeny depending on the genetic background and can speed up the maize breeding process tremendously, especially when combined with genome-editing techniques such as CRISPR-Cas9 (Kalinowska et al., 2019; Jacquier et al., 2021; Impens et al., 2023).

### Maize germplasm diversity

Although maize has gone through some evolutionary bottlenecks during domestication and directional selection, the maize breeding germplasm still has standing variability with a wide phenotypic diversity, nutritional qualities and resistance against (a)biotic stresses. Two groups in particular are very popular in maize breeding in North America and Europe: the Northern Flints and Southern Dents (Troyer, 1999; Barrière et al., 2005). The Northern Flints are described as cold tolerant, early flowering and mature, having long slender ears with undented round kernels. The Southern Dents are more heat tolerant with later maturity, being taller, higher-yielding, and having wide ears with higher kernel rows and dented rectangular kernels. Crosses between the two groups provide better adapted and higher-yielding varieties in a range of environments (Troyer, 1999).

The success of highly adapted maize varieties is the result of ingenious farmers who contributed to population improvement and developed prominent OPVs, including, but not limited to, the Reid Yellow Dent, Lancaster Sure Crop, Minnesota 13, Leaming Corn, Northwestern Dent and Longfellow Flint (Sprague and Dudley, 1988). This heterogeneous group of OPVs was classified by breeders into heterotic groups. The major heterotic groups present in current maize breeding programs can be divided into the female heterotic groups, showing high kernel yield and smaller tassels, and the male heterotic groups, with more pollen and longer pollen shedding duration. Heterotic groups PA and Stiff Stalk (represented by inbreds such as B73 and B104) are mainly used as female heterotic groups, while PB, SPT, Non-Stiff Stalk (represented by inbreds such as Mo17, Oh43 and H99) and Iodent (represented by inbreds such as PH207) are predominantly used as male heterotic groups (Li et al., 2022). These heterotic groups continually expand by the creation of new inbred lines and the occasional introgression of exotic germplasm. Phenotypic and genetic evaluation of recent inbred lines from various heterotic groups revealed that advantageous alleles for traits accumulate within the heterotic groups during selection. Some traits evolved divergently between the female and male heterotic groups (e.g. tassel height, kernel weight) and other convergently (e.g. stress tolerance) (Li et al., 2022). Notably, some alleles showing fixation within specific heterotic groups remain genetically heterogenous between heterotic groups, potentially contributing to heterosis upon crossing inbred lines from different groups (Gerke et al., 2015; Li et al., 2022). Maintaining or even increasing allelic diversity among heterotic groups is key for maize improvement, with novel breeding techniques offering potential acceleration of this process (see later).

Allelic diversity occurs spontaneously in germline cells and is derived from natural processes such as UV radiation, errors in DNA replication during cell division or in DNA repair after breakage, or via transposable elements (see later) (Martínez-Fortún et al., 2022). Studies have estimated the spontaneous mutation rate in maize to be 2.2 to 3.9 x 10^-8^ per site per generation (Yang et al., 2017b). The exploitation of spontaneous mutations in breeding has led to variations in *in vivo* NDF digestibility in hybrids, ranging between 36 and 60% (Méchin et al., 2000; Barrière et al., 2004a; Barrière et al., 2009b). Lignin is a major contributor to this variation in cell wall degradability. Although the exact genetic cause for the variation in lignin content among different genotypes is often unclear, quantitative trait loci (QTL) studies have shown that various loci collectively contribute to this trait (Barrière et al., 2015). However, exceptions exist where the genetic cause of certain historical maize lignin mutants turned out to be monogenic. These lignin mutants typically show a *brown midrib* (*bm*) phenotype, facilitating their study due to its easy-to-see phenotype. However, it was not until the 90’s that the genetic cause for the *bm* phenotype and its associated increase in digestibility were elucidated (see later).

### Advances in novel breeding techniques in maize

Conventional breeding happens in a relatively uncontrolled manner. The breeder chooses and crosses parental plants but the results are unpredictable at the phenotypic and genetic level (Wieczorek and Wright, 2012). Additionally, crossing and backcrossing of the hybrids to obtain elite lines is a laborious and time-consuming process. Marker-assisted selection accelerates the prediction and selection process in maize breeding, facilitating the identification of varieties with desired traits such as improved digestibility (Barrière et al., 2016; López-Malvar et al., 2019; Vinayan et al., 2021). Although conventional breeding relies on genetic diversity found in commercial varieties and landraces, this diversity can sometimes be limited. Consequently, modern biotechnological tools allowing to alter the plant’s DNA provide valuable alternatives (Hamdan et al., 2022). To mimic the process of random mutagenesis and increase genetic variation, the concept of *mutation breeding* was developed [reviewed by Ma et al. (2021)]. Mutation breeding is based on introducing mutations via radiation or chemical mutagens. Mutant populations can either be screened phenotypically, or by the use of techniques such as TILLING (Targeting Induced Local Lesions IN Genomes) that allow identifying specific mutations in genes of interest (Till et al., 2004). Another mutagenesis approach involves the use of *Mutator* (*Mu*) and *Activator* (*Ac*) lines. *Mu* transposons, often referred to as ‘jumping genes’, have the ability to randomly transpose within the genome. When activated by *Ac* genes, *Mu* transposons jump to new locations, thereby increasing the genetic variation (Bennetzen et al., 1993; May et al., 2003; Marcon et al., 2020).

In the 1990’s, another milestone in agriculture was reached with the development of transgenic breeding techniques, utilizing recombinant DNA technology and the ability of *Agrobacterium tumefaciens* to transfer DNA into host genomes to produce herbicide- and pest-resistant maize (Woo et al., 1997; Lundquist and Walters, 2001). RNA interference (RNAi) is a gene mechanism that allows to downregulate an endogenous gene by introducing part of that same gene in reverse orientation leading to double-stranded RNA that is degraded by the cellular machinery (Lindbo, 2012). Although currently no commercial RNAi lines for maize are available on the market, RNAi has been used to downregulate genes involved in lignification to study their effect on cell wall composition and degradability (see later). Although both mutation breeding approaches and transgenic breeding techniques modify the plant’s genome, there is a big difference between the two methods. Mutation breeding involves the introduction of mutations in the plant’s genome to achieve the desired traits, while recombinant DNA technology introduces foreign genes to impart new traits to the species.

### The CRISPR-Cas revolution

Unlike the mutation techniques mentioned above, which may introduce unpredictable, random and unwanted changes, the use of sequence-specific nucleases enables the editing of a pre-defined DNA sequence in the host plant by introducing a double stranded break (DSB) at or near the target site. Subsequently, the DNA repair machinery induces errors, leading to a mutation (Hamdan et al., 2022). The first sequence-specific nucleases used for genome editing in maize were zinc finger nucleases and transcription activator-like effector nucleases (Shukla et al., 2009; Liang et al., 2014). These techniques were successful but relatively laborious, because protein engineering was necessary to adjust the enzyme for every new target gene (Bortesi and Fischer, 2015). More recently, CRISPR-Cas [clustered regularly interspaced short palindromic repeats (CRISPR)-CRISPR associated proteins (Cas)] has revolutionized the field by enabling precise gene editing without introducing exogenous DNA in the final product (Van Vu et al., 2022). Hence, the emphasis has shifted towards gene editing techniques, with legislation showing a more favorable stance towards gene editing as compared to its transgenic counterpart (Wang et al., 2023; Stokstad, 2024).

The CRISPR-Cas system uses a DNA nuclease and a guide RNA to introduce a DSB, which is repaired via the error-prone non-homologous end-joining (NHEJ) or the error-free homologous directed repair (HDR) pathways (Xue and Greene, 2021). NHEJ often introduces short insertions or deletions (indels), leading to a gene knock-out (loss-of-function) or truncated proteins (Piatek et al., 2018). However, the mutation outcome after NHEJ is not predictable. This urged for the development of more precise editing techniques such as base and prime editing, both of which have been successfully demonstrated in maize (Jiang et al., 2020; Li et al., 2020). HDR is an alternative repair pathway to precisely restore the DSB using a DNA template derived from a homologous chromosome or to introduce a genetic modification using an artificial DNA repair template (Piatek et al., 2018). Despite the potential of HDR for gene replacement or gene insertion, its inefficiency limits its exploitation for crop improvement (Hamdan et al., 2022). Various studies have used this technology for diverse purposes, e.g. reducing smut susceptibility, introducing herbicide resistance, enhancing grain yield and developing more drought-tolerant maize varieties (Svitashev et al., 2015; Shi et al., 2017; Pathi et al., 2020; Liu et al., 2021a). Currently, there are no published CRISPR mutants affecting the lignin biosynthesis in maize. However, a comprehensive list of CRISPR applications in maize is available on EU-SAGE (http://www.eu-sage.eu).

Beyond single-gene mutagenesis, CRISPR-Cas9 also allows for simultaneous editing of numerous genes, including members of the same gene family. A notable multiplex genome editing approach, BREEDIT, was developed to rapidly generate a collection of multiplex edited plants. This method employs a single construct that simultaneously targets up to twelve genes, followed by self-pollination or crossing to achieve even higher order mutants. This strategy enables identification of promising gene combinations that can later be used in breeding programs (Lorenzo et al., 2023).

### CRISPR-Cas delivery strategies in maize

The delivery of plasmid DNA encoding a CRISPR-Cas construct in maize primarily relies on *Agrobacterium*-mediated transformation or biolistic delivery. The major advantage of *Agrobacterium*-mediated T-DNA delivery is its ability to integrate a single or low copy number of relatively large DNA fragments (up to 150 kb) into the plant’s genome (Frame et al., 2006; Yadava et al., 2017). Biolistics or particle bombardment is a genotype-independent T-DNA delivery method that physically breaches the plant cell with gold or tungsten particles coated with an expression vector, DNA fragments or ribonucleoprotein complexes (Yadava et al., 2017; Liang et al., 2019). Biolistics also presents some challenges such as the introduction of multiple copies and complex integration events of the vector (Jackson et al., 2013). Therefore, *Agrobacterium*-mediated transformation is generally the method of choice for maize transformation (Peterson et al., 2021). However, it is important to note that genotype-associated recalcitrance is also related to the tissue culture procedure, explant material and the *Agrobacterium* strain used (Yassitepe et al., 2021). Significant innovations have been made to overcome this genotype-associated recalcitrance, with one advancement being the codelivery of the morphogenetic regulators *BABY BOOM* (*BBM*) and *WUSCHEL* (*WUS*). The co-expression of *BBM/WUS* induces somatic embryogenesis, resulting in improved transformation efficiency (Lowe et al., 2016). For example, the transformation frequency has increased from 0% up to 15% in the B73 inbred line, known to be highly recalcitrant towards transformation (Mookkan et al., 2017). Although the codelivery of these morphogenic regulators has also increased the transformation efficiency of the B104 inbred line, continuous expression of *BBM/WUS* leads to pleiotropic developmental effects and sterility (Aesaert et al., 2022), which can be mitigated using gene excision systems such as CRE/loxP and/or inducible promoters (Lowe et al., 2016; Mookkan et al., 2017; Aesaert et al., 2022).

**Figure 2:**
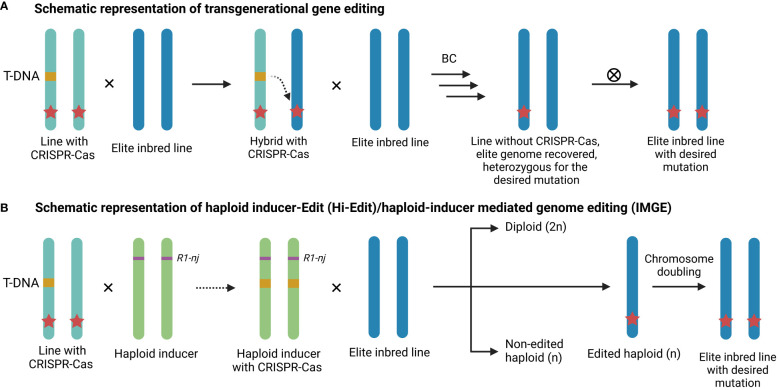
Schematic representation of transgenerational gene editing. **(A)** A maize plant containing a CRISPR-Cas T-DNA (orange) is crossed with an elite inbred line, resulting in an edited allele (red star) in the elite inbred line. The T-DNA free plants are retained and the elite background is restored via multiple rounds of backcrossing (BC). Finally, elite inbreds with homozygously edited alleles are screened for after selfing (⊗). **(B)** A CRISPR-Cas construct is introgressed or transformed into a haploid inducer, which is crossed with an elite inbred line and the edited haploids are identified. An elite inbred line homozygous for the edit is obtained after chromosome doubling.

Although CRISPR-based genome editing holds great potential in plant breeding, the genotype-associated recalcitrance mentioned earlier often limits its large-scale application for the development of new commercial maize lines. Maize lines such as Hi-II and B104 are amenable to the standard transformation protocol, however these lines are usually not suitable for commercial applications (Hernandes-Lopes et al., 2023). Moreover, commercial maize varieties are typically hybrids derived from a cross between distinct parental elite inbred lines. Consequently, introducing traits requires both parental elite inbred lines to be edited. To bypass the transformation procedure and thus the genotype-associated recalcitrance, transgenerational gene editing presents an alternative method to deliver the CRISPR-Cas machinery in elite inbred lines (Li et al., 2017; Wang et al., 2018). In short, a transgenic maize plant containing a CRISPR-Cas9 T-DNA is crossed with a recalcitrant genotype (**Figure 2A**). Within the resulting hybrid, CRISPR-Cas can then edit the target gene *in trans*. The advantage of this approach is that mutations are introduced in the recalcitrant background without the need for introgression of an allele derived from another variety, thus avoiding linkage drag (Impens et al., 2022). Nonetheless, multiple backcrosses are still required to restore the elite background, which is both a time-consuming and laborious process (Wolter et al., 2019). For example, maize plants transformed with a CRISPR-Cas construct targeting the *LIGULELESS1* (*LG1*) gene were crossed with a recalcitrant elite inbred line, resulting in an *in trans* mutation frequency of 20% in the F1 generation (Li et al., 2017). Subsequent rounds of marker-assisted backcrossing were then conducted to recover the elite background. In another example, the *GRANULE BOUND STARCH SYNTHASE I* (*GBSS I*) or *Wx* locus was *trans*-edited in two parental lines to rapidly generate a single hybrid *waxy* maize (Qi et al., 2020). Variations of transgenerational gene editing, such as the HI-edit or HI-mediated genome editing (IMGE) methods (see **Figure 2B**), have been developed in maize (Wang et al., 2018; Kelliher et al., 2019). The HI-Edit/IMGE methods involve transient expression of the CRISPR construct from the paternal HI line to edit the maternal genome *in trans*, followed by the elimination of the paternal genome in the zygote phase (Yassitepe et al., 2021). Subsequently, the edited haploid progeny undergoes artificial chromosome doubling to produce transgene-free doubled haploids (Wang et al., 2018; Kelliher et al., 2019). However, efficiencies remain low because modern HIs typically produce between 7 to 16% haploids and only 2 to 4% of the haploids are edited *in trans*. Consequently, less than 1% of the progeny are edited haploids (Wang et al., 2018; Kalinowska et al., 2019; Kelliher et al., 2019; Impens et al., 2022).

## Part II. Optimization of maize lignocellulosic biomass for agricultural and industrial applications

Increasing the nutritional value of feed and replacing fossil fuels to produce bio-based products have been the driving forces behind the increased interest in cell wall biosynthesis and its enzymatic degradation. Primary targets in maize breeding for feed purposes have been related to grain yield and whole plant biomass yield. A comparison of ten different forage maize cultivars released between 1972 and 2020 revealed an average annual yield increase of 0.13 ton dry matter (DM)/ha (Taube et al., 2020). Comparable trends in yield increase were observed in forage hybrids released between 1950 and 1980 (0.07 ton DM/ha) and from 1991 to 2003 (0.18 ton DM/ha) (Barrière et al., 1987; Luciani, 2004). However, while substantial progress has been made in improving yield, improvements in forage quality remain modest. Barrière et al. (2006) reported even a decrease in cell wall digestibility during the breeding period of forage maize in Europe from 1958 to 2002, and subsequent research by Taube et al. (2020) found no significant increase in cell wall digestibility among cultivars released between 1972 and 2020. Although a range of naturally occurring genetic variants and induced mutants with increased biomass digestibility have been identified, including the aforementioned *bm* mutants having defects in lignin biosynthesis, it has been difficult to exploit them in modern cultivars due to their pleiotropic effects (prone to lodging and reduced yield) (Vignols et al., 1995; Halpin et al., 1998; Chen et al., 2012b; Tang et al., 2014; Li et al., 2015; Xiong et al., 2019). Here it is important to note that not all maize lines with altered lignin show a *bm* phenotype (Tamasloukht et al., 2011; Fornalé et al., 2012; Li et al., 2013; Marita et al., 2014), implying that numerous lignin-modified maize mutants may have been overlooked in breeding programs that primarily screened for this characteristic *bm* phenotype. Therefore, research has become increasingly focused on investigating mutants and transgenic plants perturbed in specific steps of the lignin biosynthesis pathway. Ideally, modifying these genes or their expression levels would reduce the biomass recalcitrance without compromising the biomass yield. Below, we describe the biosynthesis of lignin and its interaction with hemicellulose, followed by an overview of mutant and transgenic maize lines with altered lignin and the resulting improvement in digestibility.

### Lignin biosynthesis

Lignin is a structural component of the cell wall that accounts for up to 17.2% of the lignocellulosic biomass in maize (Zhu et al., 2005). Lignin offers support to the cell wall, facilitates water transport and protects the carbohydrates from being digested by pathogens and insects. It is a complex aromatic heteropolymer made from phenolic monomers that are biosynthesized in the cytosol and translocated to the apoplast prior to polymerization via oxidative combinatorial coupling (Boerjan et al., 2003; Dixon and Barros, 2019; Vanholme et al., 2019). The traditional monolignols are *p*-coumaryl alcohol, coniferyl alcohol and sinapyl alcohol, that give rise to the *p*-hydroxyphenyl (H), guaiacyl (G) and syringyl (S) units in the lignin polymer, respectively (Boerjan et al., 2003; Ralph et al., 2004; Vanholme et al., 2010; Dixon and Barros, 2019; Vanholme et al., 2019). In addition, maize lignin also incorporates the flavonoid tricin, coniferaldehyde, ferulic acid and acylated monolignols such as coniferyl acetate, coniferyl *p*-coumarate, sinapyl *p*-coumarate, coniferyl ferulate and sinapyl ferulate (Lu and Ralph, 1999; Grabber et al., 2008; Ralph et al., 2008; Hatfield et al., 2009; Marita et al., 2014; Lan et al., 2015; Vanholme et al., 2019). Lignin is linked to hemicelluloses through coupling with ferulate moieties that are esterified on the arabinoxylan (Hatfield et al., 2017).

**Figure 3:**
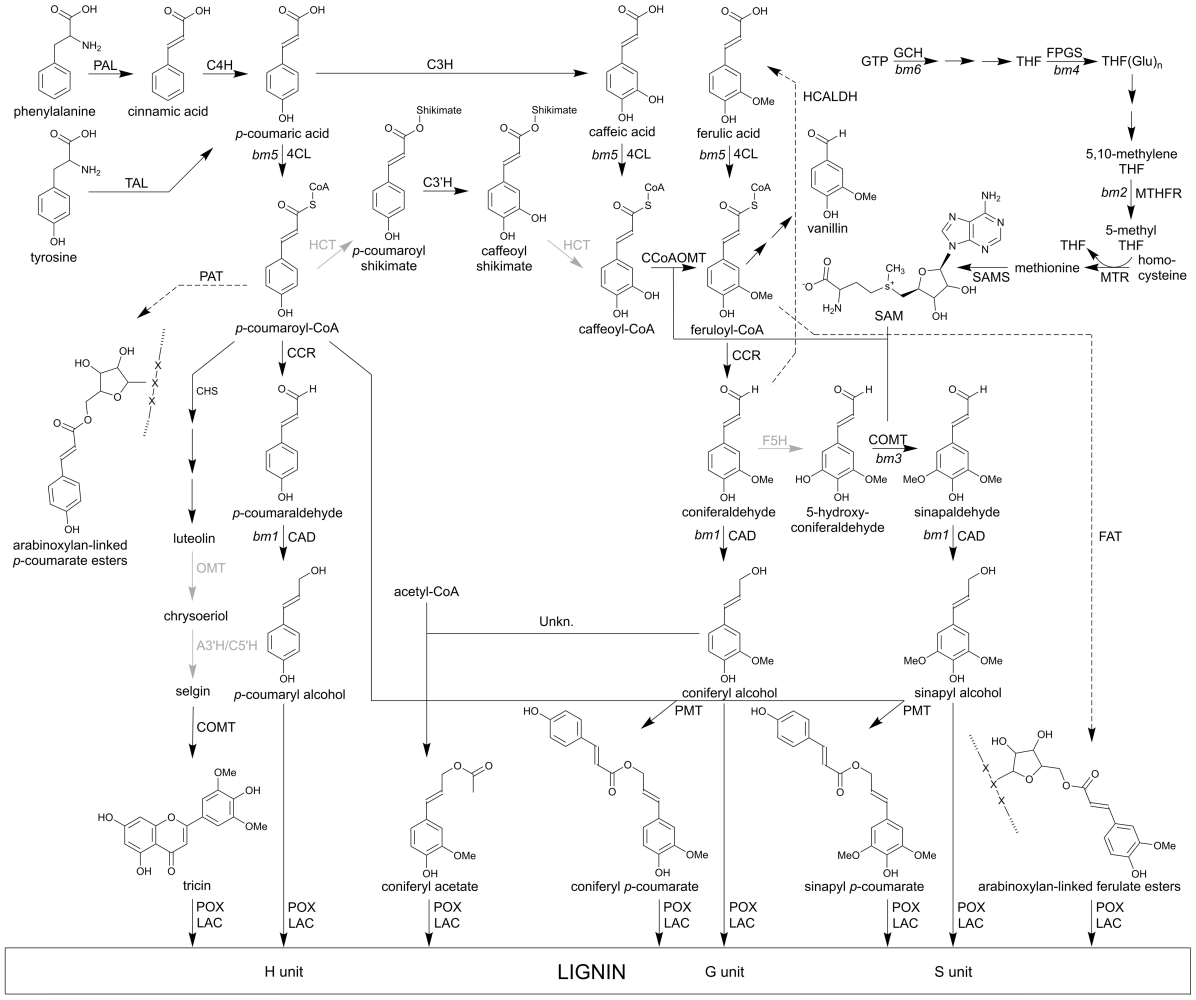
The main pathway of the lignin monomers in maize. Many metabolites are derived from the phenylpropanoid biosynthesis pathway. Here, only the routes to lignin monomers are shown. Solid arrows represent enzymatic steps evidenced by at least *in vitro* activity, dashed arrows indicate suggested conversions. Successive arrows indicate two or multiple metabolic conversions. The enzymatic conversions indicated in black are proven in maize, those that are shown in gray occur with certainty in grasses. PAL, PHENYLALANINE AMMONIA-LYASE; TAL, TYROSINE AMMONIA LYASE; C4H, CINNAMATE 4-HYDROXYLASE; C3H, *p*-COUMARATE 3-HYDROXYLASE; 4CL, 4-COUMARATE:CoA LIGASE; HCT, *p*-HYDROXYCINNAMOYL-CoA:QUINATE/SHIKIMATE *p*-HYDROXYCINNAMOYLTRANSFERASE; C3’H, *p*-COUMAROYL SHIKIMATE 3’-HYDROXYLASE; CCoAOMT, CAFFEOYL-CoA O-METHYLTRANSFERASE; CCR, CINNAMOYL-CoA REDUCTASE; F5H, FERULATE 5-HYDROXYLASE; COMT, CAFFEIC ACID O-METHYLTRANSFERASE; CAD, CINNAMYL ALCOHOL DEHYDROGENASE; HCALDH, HYDROXYCINNAMALDEHYDE DEHYDROGENASE; PMT, *p*-COUMAROYL-CoA MONOLIGNOL TRANSFERASE; CHS, CHALCONE SYNTHASE; A3’H/C5’H, APIGENIN 3’-HYDROXYLASE/CHRYSOERIOL 5’-HYDROXYLASE; POX, PEROXIDASE; LAC, LACCASE; PAT, *p*-COUMAROYL-CoA ARABINOFURANOSE TRANSFERASE; FAT, FERULOYL ARABINOFURANOSE TRANSFERASE; GTP, guanosine triphosphate; GCH1, GTP CYCLOHYDROLASE; THF, tetrahydrofolate; FPGS, FOLYLPOLYGLUTAMATE SYNTHASE; MTHFR, METHYLENETETRAHYDROFOLATE REDUCTASE; MTR, 5-METHYLTETRAHYDROFOLATE HOMOCYSTEINE METHYLTRANSFERASE; SAMS, S-ADENOSYLMETHIONINE SYNTHETASE; SAM, S-adenosyl-L-methionine. *bm*; *brown midrib* mutants.

The elucidation of the lignin biosynthetic pathway in maize is a work in progress. **Figure 3** summarizes the latest insights into the general and the monolignol-specific pathway and compiles which enzymatic steps have been proven to occur in maize and which are based on findings in other grass species. Lignin is synthesized in a series of enzymatic steps starting from phenylalanine and tyrosine (Boerjan et al., 2003; Vanholme et al., 2010, 2019; Barros and Dixon, 2020). The first step of the general phenylpropanoid pathway is the conversion of phenylalanine by PHENYLALANINE AMMONIA LYASE (PAL) to cinnamic acid. Next, cinnamic acid is hydroxylated by CINNAMIC ACID 4-HYDROXYLASE (C4H) to form *p*-coumaric acid (Ôba and Conn, 1988). PALs in grasses, including maize, can be bifunctional and also have TYROSINE AMMONIA LYASE (TAL) activity that allows the conversion of tyrosine into *p*-coumaric acid (Rösler et al., 1997; Barros et al., 2016). Subsequently, *p*-coumaric acid is converted into *p*-coumaroyl-CoA through 4-COUMARATE:CoA LIGASE (4CL) (Yun et al., 2005; Xiong et al., 2019). Next, *p*-coumaroyl-CoA is esterified into its corresponding shikimic or quinic ester derivative catalyzed by *p*-HYDROXYCINNAMOYL-CoA: QUINATE/SHIKIMATE *p*-HYDROXYCINNAMOYLTRANSFERASE (HCT). This enzymatic conversion has not yet been proven to occur in maize, although it has been identified in other grass species like Brachypodium, switchgrass and sorghum (Shadle et al., 2007; Walker et al., 2013; Escamilla-Treviño et al., 2014; Serrani-Yarce et al., 2021). In turn, *p*-coumaroyl shikimate is hydroxylated by *p*-COUMAROYL-CoA 3’-HYDROXYLASE (C3’H) to produce caffeoyl shikimate. Currently, two C3’H-encoding genes, namely *C3’H1* and *C3’H2*, have been found in maize (Barrière et al., 2007; Guillaumie et al., 2007); the role of C3’H1 in lignin biosynthesis has already been demonstrated through reverse genetics (Fornalé et al., 2015). Caffeoyl shikimate is then further esterified by HCT into caffeoyl-CoA (Serrani-Yarce et al., 2021). Alternatively to the biosynthetic route via 4CL, HCT, C3’H and again HCT, *p*-coumaric acid can also be converted into caffeoyl-CoA via a two-step pathway; hydroxylation by COUMARATE 3-HYDROXYLASE (C3H) (Barros et al., 2019) and ligation to CoA by 4CL. In the last step of the general phenylpropanoid pathway, caffeoyl-CoA is methylated by CAFFEOYL-CoA O-METHYLTRANSFERASE (CCoAOMT) into feruloyl-CoA (Wu et al., 2018).

The first committed enzyme of the monolignol-specific pathway is CINNAMOYL-CoA REDUCTASE (CCR) that converts hydroxycinnamoyl-CoA esters (*p*-coumaroyl-CoA and feruloyl-CoA) into their corresponding hydroxycinnamaldehydes (*p*-coumaraldehyde and coniferaldehyde) (Pichon et al., 1998). Coniferaldehyde can be converted into 5-hydroxyconiferaldehyde through FERULATE 5-HYDROXYLASE (F5H) (Kim et al., 2006; Wu et al., 2019). The maize genome encodes two *F5H* genes (Guillaumie et al., 2007), of which *F5H1* was previously described as being associated with a QTL for digestibility by Puigdomenech et al. (2001), but no functional proof was given for either of the two genes through reverse genetics. In turn, 5-hydroxyconiferaldehyde is methylated by CAFFEIC ACID O-METHYLTRANSFERASE (COMT) into sinapaldehyde (Piquemal et al., 2002). Lastly, CINNAMYL ALCOHOL DEHYDROGENASE (CAD) catalyzes the conversion of the hydroxycinnamaldehydes into the traditional monolignols, *p*-coumaryl, coniferyl and sinapyl alcohol (Halpin et al., 1998). The respective hydroxycinnamyl alcohols can function either directly as lignin monomers or as substrates for acyltransferase reactions, resulting in γ-*O*-acylated monolignol conjugates that can also be incorporated into the lignin polymer. In maize, similar to other grasses, coniferyl alcohol is acylated with acetate via an enzyme yet to be identified, and primarily sinapyl alcohol is acylated with *p*-coumaric acid (*p*CA) by *p*-COUMAROYL-CoA: MONOLIGNOL TRANSFERASE (PMT) (Withers et al., 2012; Marita et al., 2014; Karlen et al., 2018). Monolignols (mainly sinapyl alcohol) can also be acylated with ferulic acid, via FERULOYL-CoA MONOLIGNOL TRANSFERASE (FMT) activity, although such conjugates are less abundant (Karlen et al., 2016; Smith et al., 2017).

### Biosynthetic routes branching from the lignin pathway

The phenylpropanoid pathway is connected with other biosynthetic routes, some of which result in other lignin monomers, such as flavonoids, or molecules that connect lignin with hemicelluloses (Vanholme et al., 2019). For example, *p*-coumaroyl-CoA is the substrate of CHALCONE SYNTHASE (CHS), the key enzyme in regulating the flux toward the biosynthesis of flavonoids, including the lignin monomer tricin. Tricin is known to be incorporated in lignin, where it acts as an initiation site for lignin polymerization (Lan et al., 2015, 2016; Eloy et al., 2017). In grass cell walls, ferulate and to a lesser extent *p*-coumarate, can acylate the hemicellulose polymer via the C5-hydroxyl of the α-L-arabinosyl side chains of glucuronoarabinoxylan (GAX). These ferulate groups originate from feruloyl-CoA and are coupled to the arabinose residue by a FERULOYL ARABINOFURANOSE TRANSFERASE (FAT) belonging to the BAHD acyltransferases (**Figure 3**) (Schmitt et al., 1991; Guo et al., 2001; de Oliveira et al., 2015; Fanelli et al., 2021; Chandrakanth et al., 2023). Feruloyl-CoA is a phenylpropanoid pathway intermediate, and thus readily available in lignifying cells.

### Polymerization

After their biosynthesis, the lignin monomers are thought to passively diffuse through the plasma membrane into the apoplast (Vermaas et al., 2019; Perkins et al., 2022). In the cell wall, the monomers are oxidized by peroxidases (POXs) and/or laccases (LACs) into their corresponding radicals (**Figure 3**) (Guillet-Claude et al., 2004; Zhao et al., 2013; Perkins et al., 2022). During lignin polymerization, these monomer radicals will undergo combinatorial coupling, resulting in a variety of chemical bonds, of which the β-aryl (8-*O*-4), resinol (8–8) and phenylcoumaran (8–5) bonds are the most frequent ones (Ralph et al., 2019). LAC4 has been described to be involved in lignification of the maize cob and to affect the ear length (Bi et al., 2024). Due to the broad substrate specificity of LACs and POXs, and the possibility of radical transfer, the ferulates present on GAX can also undergo oxidation and radical coupling, resulting in the formation of dehydrodiferulate dimers or oligomers, which crosslink the polysaccharide chains. Additionally, ferulates are also crosslinked to lignin polymers, forming covalently linked carbohydrate–lignin complexes (de Oliveira et al., 2015; Hatfield et al., 2017; Terrett and Dupree, 2019). Thereby, ferulates act as nucleation sites for lignin formation (Buanafina, 2009; Courtial et al., 2013; Hatfield et al., 2017; Terrett and Dupree, 2019). Furthermore, free ferulic acids can also be incorporated into the lignin polymer by 8-*O*-4 crosslinking, allowing new branching points with biphenyl structures to be formed (Ralph et al., 2008).

### Upstream regulation of the lignin pathway

The transcriptional regulation of genes involved in lignin biosynthesis is tightly controlled by transcription factors of the MYB and NAC families (Barrière et al., 2015) (Zhao and Dixon, 2011; Zhong and Ye, 2015). But so far, few transcription factors have been evaluated by reverse genetics in grasses (Wang et al., 2016; Bhatia et al., 2017). Expression profiling of Arabidopsis overexpression lines has shown that maize ZmMYB31 and ZmMYB42 both act as repressors of *COMT* and potentially other lignin biosynthetic genes (Fornalé et al., 2006; Sonbol et al., 2009; Fornalé et al., 2010). It was later discovered that ZmMYB69 acts as an activator of *ZmMYB31* and *ZmMYB42* expression, and thus a repressor of lignin biosynthesis (Qiang et al., 2022). In contrast, maize ZmMYB46 is described as a master switch to activate cell wall biosynthesis, including cellulose, hemicellulose and lignin biosynthesis, based on overexpression experiments in Arabidopsis (Zhong et al., 2011). Furthermore, there is evidence that both ZmMYB5 and ZmMYB152 (which are also called ZmMYB148 and ZmMYB111, respectively) are activators of lignin biosynthesis, but their function has not yet been evaluated *in planta* (Zhang et al., 2016; Yang et al., 2017a). Based on overexpression in Brachypodium and maize, also ZmMYB167 has been identified as an activator of lignin biosynthesis (Bhatia et al., 2019). NAC transcription factors regulate the expression of downstream MYBs (Xiao et al., 2018). Specifically, NAC SECONDARY WALL THICKENING PROMOTING FACTOR 3 (NST3) and NST4 regulate the expression of, amongst others, *MYB109*, *MYB128* and *MYB149*, and enhance cell wall thickening (Xiao et al., 2018; Ren et al., 2020). Even though NST3 and NST4 are shown to activate lignin biosynthesis in maize, the transcriptional cascades involved are not yet described (Xiao et al., 2018; Ren et al., 2020).

### Lignin mutants

The availability of genomic information and insights into the lignin biosynthetic pathway have made it possible to make perturbations in this pathway to modify and steer the biosynthesis of the monomers or the structure of the polymer itself (Mottiar et al., 2016; Ralph et al., 2019). **Table 1** provides an overview of maize lines with altered lignin amount and composition, and their cell wall degradability and biomass yield. It should be noted that variation in the genetic background in which the various mutations were studied hinders straightforward comparisons of the specific effects of a particular perturbation (Chabbert et al., 1994; Marita et al., 2003). Indeed, different inbred lines already differ significantly in their lignin content and forage quality (Lundvall et al., 1994). Hence, mutations in different genetic backgrounds may result in different phenotypes. In addition, often different mutant alleles, e.g. knock-out and weak alleles, have been described for a given gene, or transgenic plants may have different degrees of downregulation of the target gene (Park et al., 2012; Barrière et al., 2013). This genetic variation is reflected in the range of mutant phenotypes associated with a single gene perturbation.

**Table 1:** List of lignin mutants in maize and the effects of the mutation on the lignin content, lignin composition, cell wall degradability and biomass yield.

Gene	Method	Growth conditions	Biomass yield	Lignin content	Lignin composition	Digestibility	Reference
*C4H3*	Antisense	Greenhouse	n.d.	Leaf: 14–17%↓	n.d.	n.d.	Abdel-Rahman and Mousa (2016)
*4CL1* (*bm5*)	Transposon	Greenhouse	WT *bm* phenotype	Stem: 10–20%↓ Midrib: WT	S/G↑, %H↑ *p*CA↓	NDFD: ~22%↑ Saccharification efficiency: ~18%↑	Méchin et al. (2014); Xiong et al. (2019)
*C3’H1*	RNAi	Greenhouse	Plant height↓ Sterility	Stem: 19–23%↓ Midrib: WT	S/G: WT, %H↑	Saccharification efficiency: ~32%↑	Fornalé et al. (2015)
*CCoAOMT1*	RNAi	Greenhouse	WT Delayed growth	Whole plant, without ear: ~22%↓	S/G↑	n.d.	Li et al. (2013)
*CCR1*	Transposon	Field	WT	Whole plant, without ear: ~12%↓	S/G↑, %H↓	NDFD: ~15%↑	Tamasloukht et al. (2011)
*CCR1*	RNAi	Greenhouse	WT Abnormalities (5%) *bm* phenotype (30%)	Stover: 7–8%↓	n.d.	Saccharification efficiency: 7–8%↑	Park et al. (2012)
*CCR1*	Transposon	Greenhouse Field	Greenhouse: WT Field: DM ~28%↑	Stem: ~20%↓	S/G↑, %H: WT %Sinapyl ferulate units↑	Saccharification efficiency: 40–53%↑	Smith et al. (2017)
*COMT* (*bm3*)	Transposon	Field	DM: 5–20%↓	Stem: 20–40%↓	S/G↓, %H↓ *p*CA↓ 5-hydroxyguaicyl units↑	n.d.	Barrière et al. (1994); Chabbert et al. (1994); Vignols et al. (1995)
*COMT*	Deletion	Field	n.d.	n.d.	n.d	n.d.	Vignols et al. (1995)
*COMT*	n.d.	Field	n.d.	Stem: 20%↓	FA↑ *p*CA: ~50%↓ 5-hydroxyguaicyl units↑	n.d.	Marita et al. (2003)
*COMT*	n.d.	Field	n.d.	Stem: ~21%↓	S/G↓, %H↓ *p*CA↓ 5-hydroxyguaicyl units↑	NDFD: ~45%↑	Barrière et al. (2004b)
*COMT*	Antisense	Greenhouse	WT *bm* phenotype	Whole plant, without ear: 25–30%↓	S/G↓,%H↓ *p*CA↓ FA↑ 5-hydroxyguaicyl units↑	NDFD: ~9%↑	Piquemal et al. (2002); Barrière et al. (2017)
*COMT*	Antisense	Greenhouse	WT *bm* phenotype	Stem: ~20%↓ Leaf: ~12%↓	n.d.	NDFD: ~5%↑	He et al. (2003)
*COMT*	Antisense	Field	*bm* phenotype Plant height: 15–30% ↓	Stem: ~10%↓	S/G↓ *p*CA↓ 5-hydroxyguaicyl units↑	NDFD: ~23%↑	Pichon et al. (2006)
*COMT*	n.d.	Greenhouse	*bm* phenotype Stem DW: ~36%↓	Stem: ~43%↓ Midrib: WT	S/G↓, %H↓	Saccharification efficiency: Stem: ~9%↑ Midrib: ~12%↓	Fornalé et al. (2017)
*CCoAOMT1*	Transposon	Greenhouse	WT	WT	%H↓	Saccharification efficiency: Stem: ~32%↑	Fornalé et al. (2017)
*COMT CCoAOMT1*	n.d. Transposon	Greenhouse	*bm* phenotype Stem DW: ~25%↓	Stem: ~38%↓ Midrib: ~33%↓	S/G↓, %H↓	Saccharification efficiency: Stem: ~23%↑ Midrib: ~16%↑	Fornalé et al. (2017)
*CAD2* (*bm1*)	n.d.	Greenhouse	n.d.	Stem: ~21%↓	S/G↑, %S↓, %G↓ *p*CA↓	NDFD: ~10%↑	Barrière et al. (1994)
*CAD2*	2 bp insertion, method n.d.	Greenhouse	*bm* phenotype WT	Stem: ~20%↓	S/G: WT FA(A_g_)↑	n.d.	Halpin et al. (1998); Barrière et al. (2013)
*CAD2*	Transposon	Greenhouse	*bm* phenotype WT	Whole plant, without ear: 6–17%↓	*p*CA↓ FA(A_g_)↑ Coniferaldehyde incorporation↑	NDFD: ~12%↑	Barrière et al. (2013)
*CAD2*	n.d.	Field	n.d.	Stem: WT	FA↓ *p*CA↓ Coniferaldehyde incorporation↑	n.d.	Marita et al. (2003)
*CAD2*	RNAi	Greenhouse Field	WT	Stem: WT Midrib: ~6%↓	S/G↓, %H↑ Coniferaldehyde incorporation: WT	Saccharification efficiency: 16–19%↑	Fornalé et al. (2012)
*CAD2*	n.d.	Field	n.d.	Stem: ~11%↓	S/G↓ FA↓ *p*CA↓ Coniferaldehyde incorporation↑	NDFD: ~21%↑	Barrière et al. (2004b)
*CAD2*	Chemically induced (EMS)	Greenhouse	*bm* phenotype	Midrib: ~5%↓	S/G↑	Saccharification efficiency: ~58%↑	Xiong et al. (2020)
*CAD2*	Transposon	Field	n.d.	Whole plant, without ear: ~18%↓	S/G: WT, %H↓ *p*CA↓ FA: WT Coniferaldehyde and sinapaldehyde incorporation↑	Saccharification efficiency: 20%↑	Liu et al. (2021b)
*CAD2*	Transposon	Greenhouse	*bm* phenotype	Leaves+stem: 24–30%↓	n.d.	n.d.	Chen et al. (2012a)
*MTHFR*1 (*bm2*)	n.d.	Field	n.d.	Stem: WT	FA↑	n.d.	Marita et al. (2003)
*CAD2* (*bm1*) *MTHFR1* (*bm2*)	n.d.	Field	*bm* phenotype	Stem: 9%↓	Coniferaldehyde and sinapaldehyde incorporation↑	n.d.	Marita et al. (2003)
*MTHFR1*	n.d.	Field	n.d.	Stem: ~17%↓	S/G↑ FA↓	NDFD: ~27%↑	Barrière et al. (2004b)
*MTHFR1*	Transposon	n.d.	*bm* phenotype	Stem: 7%↓	S/G↑	n.d.	Tang et al. (2014)
*MTHFR1*	Transposon	Greenhouse	WT *bm* phenotype	Midrib: WT	S/G↑, %H↑	Saccharification efficiency stem: ~58%↑	Wu et al. (2018)
*FPGS* (*bm4*)	n.d.	Field	n.d.	Stem: WT	FA↑	n.d.	Marita et al. (2003)
*FPGS*	n.d.	Field	n.d.	Stem: ~13%↓	S/G↑ *p*CA ↓ FA ester: ~6%↑ FA ether: ~19%↓	NDFD: ~43%↑	Barrière et al. (2004b)
*FPGS*	Transposon	Field	WT *bm* phenotype	Stem: 10–14%↓	S/G↑	n.d.	Li et al. (2015)
*GCH1* (*bm6*)	Transposon	n.d.	Plant height: ~6%↓	Stover: ~9%↓	n.d.	NDFD: ~3%↑	Chen et al. (2012a); Leonard et al. (2021)
*PMT*	RNAi	Greenhouse	n.d.	Stem: WT Leaf: WT	S/G↓ *p*CA↓	n.d.	Marita et al. (2014)
*POX3*	Transposon	Greenhouse	n.d.	n.d.	n.d.	NDFD: ~12%↑	Guillet-Claude et al. (2004)
*CHS*	RNAi	Greenhouse	Total DM: 18–32%↑ Leaf DM: 25–27%↑	Stem: WT Leaf: ~27%↑	Stem: S/G: WT, %H↓ Tricin: 100%↓ Leaf: S/G: WT, %H: WT Tricin: 98%↓	Saccharification efficiency leaf: 39%↓	Eloy et al. (2017)
Unknown gene (*sfe)*	Transposon	Field	Plant height: ~4%↑ Biomass: ~14%↑	Stem: 5–19%↓ Sheat: 3–10%↓	Stem: S/G↑ FA↓ *p*CA↓ Sheat: FA↓ *p*CA↓	NDFD: Stem: ~3%↑ Sheat: ~4%↑	Jung and Phillips (2010)
*MYB69*	Overexpression	Greenhouse	Plant height ↓	Stem: ↓	n.d.	Saccharification efficiency: ↑	Qiang et al. (2022)
*MYB69*	CRISPR	Greenhouse	WT	Stem: ↑	n.d.	n.d.	Qiang et al. (2022)
*MYB167*	Overexpression	Greenhouse	WT	Stem: 4–13%↑	*p*CA↑ FA↑	Saccharification efficiency: WT	Bhatia et al. (2019)
*NST3*	Overexpression	Greenhouse	Plant height ↓	Stem: ↑	n.d.	n.d.	Xiao et al. (2018)
*NST3*	Antisense	Greenhouse	Plant height ↓ Arrested growth, up-curled leaves	Stem: ↓	n.d.	n.d.	Xiao et al. (2018)
*NST4*	Antisense	Greenhouse	Plant height ↓ Tubular leaves	Stem: ~70%↓	n.d.	n.d.	Xiao et al. (2018)

The full-length gene names corresponding to the abbreviations are provided in the legend of **Figure 3**. For lignin composition, the %H is expressed on the total amount of monomers released from the lignin (H+G+S), and *p*CA and FA refer to the relative change in their levels expressed per total cell wall, *p*CA; *p*-coumaric acid, FA; ferulic acid, DM, dry matter; NDFD, neutral detergent fiber digestibility; *bm*, brown midrib; FA(A_g_), marker for ferulic acid incorporation as monomer in lignin; ↑, increase; ↓, decrease; WT, equals to wild-type levels; n.d., not determined.

Maize lines have been described that are mutated or downregulated in specific genes or gene family members of the general phenylpropanoid pathway, such as *C4H3*, *4CL1*, *C3’H1* and *CCoAOMT* (**Table 1**). Downregulation of *C4H3* results in a decrease in lignin content of 14 to 17%, but no cell wall degradability tests have been reported for these lines (Abdel-Rahman and Mousa, 2016). The *bm5* mutation that affects the *4CL1* gene results in a 10 to 20% decrease in lignin content without affecting the biomass yield (Méchin et al., 2014; Xiong et al., 2019). The neutral detergent fiber digestibility (NDFD) and saccharification efficiency of *bm5* are increased on average by 18% and 22%, respectively (Xiong et al., 2019). Furthermore, the *bm5* mutants show a 20 to 30% reduction in *p*CA ester levels and a 5- to 20-fold increase in the incorporation of ferulic acid into the lignin polymer (Méchin et al., 2014). Downregulation of *C3’H1* by RNAi in maize results in a tendency towards a lower lignin content in stem tissue, while lignin content remains unchanged in the midrib (Fornalé et al., 2015). Additionally, the lignin composition in stem tissue shifts to an increased proportion of H units, a decreased frequency of S units and a tendency towards an increased proportion of G units. Moreover, although the *in vitro* digestibility is increased by 32%, the *C3’H1*-RNAi plants suffer from a growth reduction and male sterility (Fornalé et al., 2015). Downregulation of *CCoAOMT1* in maize results in a decrease of 22% in lignin content and an increase in the S/G ratio. Besides a slight growth delay, the *CCoAOMT1*-RNAi lines are indistinguishable from wild-type (WT) plants (Li et al., 2013).

Maize lines with mutations in the monolignol-specific pathway have been reported for *CCR*, *COMT* and *CAD* (**Table 1**). Studies have shown that *CCR*-deficient plants have a lower lignin content, which translates into a significant increase in saccharification efficiency. For example, a *Mu* insertion in the first intron of the *CCR1* gene results in a 31% reduction in *CCR1* expression, leading to a 12% reduction in Klason lignin content, accompanied by an increase in the S/G ratio as well as a decreased frequency of H lignin units. These modifications are associated with a 15% improved *in vitro* digestibility. The *ccr1* mutants do neither have a biomass yield penalty, nor a *bm* phenotype, when grown in the greenhouse (Tamasloukht et al., 2011). Similarly, when *CCR1* is downregulated via an RNAi strategy, a reduction in Klason lignin content of 7 to 8% is accompanied by a 7 to 8% increased enzymatic conversion to fermentable sugars upon an ammonia fiber expansion pretreatment. However, six out of the twenty generated RNAi lines have a *bm* phenotype and a normal biomass yield, while 5% of the RNAi lines show stunted growth with curly leaves and aborted early flowering (Park et al., 2012). A third study examined the lignin content, composition and saccharification efficiency of a maize line with a *Mu* insertion in the fourth exon of *CCR1*. These maize lines have an approximately 20% lower lignin amount and show an up to 53% increased digestibility in a limited-extend digestibility test. These mutants have an increased S/G ratio and release more sinapyl ferulate units by DFRC relative to WT plants. These maize plants have neither a yield penalty nor a *bm* phenotype when grown in the greenhouse. Furthermore, the latter mutants have a 16% reduction in seed weight and an overall 28% increase in total biomass yield when grown in the field (Smith et al., 2017). Presumably, the differences in the phenotypes between the RNAi and stable *ccr1* mutants are due to the possibility that in the RNAi lines, multiple *CCR* gene family members are simultaneously downregulated, resulting in further reductions in lignin content as compared to the lines where only *CCR1* is mutated. These data also imply that one or more *CCR* gene family members other than *CCR1* have a role in determining lignin content, and these gene family members still need to be pinpointed.

The *bm3* mutants in maize have a defective *COMT* gene and exhibit an improved *in vitro* digestibility by up to 45% (Vignols et al., 1995). These mutants also have an average reduction of 20% in lignin content with a striking decrease in S units. Moreover, the esterified *p*CA content is consistently decreased, while in some instances, an increase in esterified ferulic acid levels is observed, but no differences in the level of crosslinking between arabinoxylans (Chabbert et al., 1994; Marita et al., 2003; Barrière et al., 2004b). Downregulation of *CAD2* leads to a reduction in lignin content ranging from 4 to 20%, depending on the particular mutation or method used. Furthermore, a reduced content of *p*CA esters is observed, accompanied by an increase in coniferaldehyde, sinapaldehyde and ferulic acid incorporation into the lignin polymer (Ralph et al., 2008; Barrière et al., 2013; Liu et al., 2021b). Additionally, *bm1* mutants, defective in *CAD2*, exhibit an improvement of up to 58% in saccharification efficiency (Xiong et al., 2020).

In addition, mutant maize lines have been described that have a reduced availability of S-adenosyl-methionine (SAM), the methyl donor for CCoAOMT and COMT (**Figure 3**). For example, *bm2* mutants show a significant reduction in *METHYLENE-TETRAHYDROFOLATE REDUCTASE 1 (MTHFR1)* expression, resulting in a 7 to 17% decrease in lignin, an increase in the S/G ratio in stalks and a 21% increase in NDFD compared to WT plants (Barrière et al., 2004b; Tang et al., 2014). Furthermore, Wu et al. (2018) reported *bm2* plants with lignin levels similar to WT but with an increase in the S/G ratio and fraction of H units. These changes lead to a notable 58% improvement in saccharification efficiency. On the other hand, the *bm4* mutation impacts FOLYLPOLYGLUTAMATE SYNTHASE (FPGS) (Cossins and Chen, 1997; Mehrshahi et al., 2010). Similar to *bm2*, *bm4* mutants accumulate 10 to 14% less lignin in the stalks, have an elevated S/G ratio and show an increase in NDFD by 43% (Barrière et al., 2004b; Li et al., 2015). The *bm6* mutant phenotype is caused by a loss-of-function mutation in *GTP-CYCLOHYDROLASE 1* (*GCH1*), encoding an enzyme functioning upstream of FPGS, where it mediates the first step in the tetrahydrofolate (THF) biosynthesis pathway. These mutants show a 6% reduction in lignin content and a 3% improvement in NDFD (Chen et al., 2012b; Leonard et al., 2021).

A transgenic line downregulated in *PMT* shows a substantial reduction in esterified *p*CA levels, accompanied by a decrease in S units (Marita et al., 2014). Furthermore, the naturally silenced *colorless* (*c2*) mutant, which is defective in the *CHALCONE SYNTHASE* gene, exhibits a 98% reduction in tricin incorporation in the leaves whereas tricin is below the detection limit in the mutant stems. Despite this, the *c2* mutant exhibits WT lignin levels in the stem, although with a reduction in H units. Conversely, in leaf tissue, the *c2* mutant shows a 27% increase in lignin content, which is accompanied by a decrease in saccharification efficiency of 39% (Eloy et al., 2017). Finally, maize lines with a defective allele of the peroxidase-encoding gene *ZmPOX3*, resulting from a MITE transposon insertion, exhibit higher forage digestibility compared to lines with similar genetic backgrounds but carrying a functional allele of *ZmPOX3* (Guillet-Claude et al., 2004).

Only a limited number of maize lines has been described with altered levels of MYB or NAC transcription factors involved in lignin biosynthesis (**Table 1**). *ZmMYB167* overexpression lines are not affected in their growth and development and exhibit an increase of 4 to 13% in lignin, 8 to 52% in *p*CA and 13 to 38% in ferulate esters in the internodes. Nevertheless, no changes in biomass recalcitrance are observed upon saccharification (Bhatia et al., 2019). Furthermore, maize lines in which *ZmMYB69* is overexpressed display a decrease in plant height, as well as in vascular bundle cell wall thickness (Qiang et al. (2022). Significantly reduced levels of lignin are observed in the *ZmMYB69* overexpression lines in comparison to the control, which is accompanied by increased saccharification efficiency. Conversely, thicker cell walls and a higher lignin content are observed in *zmmyb69* loss-of-function lines, generated via CRISPR/Cas. These plants do not show any visible growth defects. In addition, both overexpression and downregulated lines were made for *ZmNST3* and *ZmNST4*. The overexpression of *ZmNST3* leads to a decrease in plant height and an increase in cell wall thickness, which is due to an increase in lignin and cellulose content in the internodes. Meanwhile, *ZmNST4* overexpression appears to be lethal. The downregulation of either *ZmNST3* or *ZmNST4* results in a reduction in stem lignification and reduced plant height (Xiao et al., 2018).

### Causes of reduced cell wall recalcitrance

Cell wall digestibility is a complex trait influenced primarily by factors such as lignin content, lignin composition and ferulate cross-linkages (Méchin et al., 2014; Torres et al., 2015b; Barrière, 2017). Plants with a lower lignin content generally have an improved digestibility, but it is not easy to discern the contribution of specific structural lignin alterations to the improvements in biomass digestibility (Halpin, 2019). For example, an increased proportion of H units may contribute to improved biomass digestibility in maize, by engendering smaller lignin polymers (Fornalé et al., 2012; Vanholme et al., 2012; Méchin et al., 2014; Fornalé et al., 2015; Wu et al., 2018). This has been achieved by suppressing *C3’H1*, but this engineering strategy additionally resulted in a reduction of total lignin (Fornalé et al., 2015). Furthermore, a positive correlation between S/G ratio and cell wall degradability in maize has been observed (Méchin et al., 2000, 2005; Zhang et al., 2011). This could likely be attributed to the different linkage type frequencies engendered by coupling of S and G units (Barrière et al., 2009a). Where lignin rich in S units is relatively linear and abundant in (8-*O*-4) ether bonds, lignin rich in G units has more carbon-carbon (8–5 and 5–5) linkages and branched structures (Ralph et al., 2008). Additionally, the observed positive correlation might also be caused by the confounding effect that S units, as compared to G units, are more prone to esterification into *p*CA esters, which have a positive effect on cell wall degradation (Méchin et al., 2000; Grabber et al., 2009; Zhang et al., 2011).

Maize lines mutant for enzymes acting upstream of coniferaldehyde (MTHFR, 4CL1, C3’H1, CCoAOMT, CCR1) show a reduction in both G and S lignin units, with a stronger decrease in G units, resulting in an increased S/G ratio, which could contribute to the increased cell wall digestibility (Barrière et al., 2004a; Tamasloukht et al., 2011; Smith et al., 2017; Xiong et al., 2020). In contrast, the decreased COMT activity in *comt* mutants strongly reduces the biosynthesis of sinapyl alcohol, resulting in a substantial decrease in the S/G ratio. The improved digestibility of *comt* mutants can primarily be attributed to their lower lignin content, while their reduced S/G lignin ratio would rather counteract the release of cell wall sugars (Fornalé et al., 2017). However, instead of S units, the lignin polymer contains 5-hydroxyguaiacyl (5-OH-guaiacyl) subunits, that give rise to benzodioxane structures, through the incorporation of 5-hydroxyconiferyl alcohol and 5-hydroxyconiferaldehyde (Marita et al., 2003). The increased presence of benzodioxane structures in the lignin polymer is thought to reduce cross-linking of lignin with cell wall carbohydrates, in this way contributing to the cell wall degradability (Weng et al., 2010; Vanholme et al., 2012).

In addition to 5-hydroxyconiferyl alcohol and 5-hydroxyconiferaldehyde, the incorporation of other intermediates from the lignin pathway can also facilitate cell wall processing. For example, a reduced CAD activity results in the integration of coniferaldehyde and sinapaldehyde into the lignin, thereby producing a lignin polymer with a higher proportion of free phenolic units (Lapierre et al., 1999; Ralph et al., 2001). Depending on the plant species and pretreatment used, incorporation of these hydroxycinnamaldehydes results in an improved cell wall processing (Fu et al., 2011; Fornalé et al., 2012; Van Acker et al., 2017; Liu et al., 2021b). In addition, reduced CAD activity also results in the incorporation of ferulic acid into the lignin polymer, presumably because its substrate coniferaldehyde is converted by HCALDH into ferulic acid (Nair et al., 2004; de Oliveira et al., 2015; Končitíková et al., 2015; Missihoun et al., 2016; Liu et al., 2021b). The incorporation of ferulic acid results – in contrast to GAX-bound ferulate moieties (see below) – in acetal bonds that are cleavable in acidic conditions (Ralph et al., 2008). However, in different lignin engineering strategies in a variety of plant species, the incorporation of ferulic acid appears to be marginal, presumably due to the low translocation efficiency of ferulic acid to the apoplast (Van Acker et al., 2013, 2014; Vermaas et al., 2019).

Considering that *p*CA is mainly esterified into sinapyl alcohol in maize, mutants with a reduced biosynthesis of sinapyl alcohol, such as *cad2* and *comt*, show a reduced amount of *p*CA conjugates (Piquemal et al., 2002; Barrière et al., 2013; Méchin et al., 2014). However, the strongest reduction in cell wall bound *p*CA esters in maize was observed by downregulating *PMT* (Marita et al., 2014). The *p*CA moieties are pending groups with free phenolic ends that make the lignin polymer more soluble in alkaline pretreatment conditions (Hatfield et al., 2017; Lapierre et al., 2021). Therefore, the increase in *p*CA conjugates, e.g. by overexpression of *PMT*, is expected to improve the saccharification efficiency of the maize biomass.

GAX-bound ferulates allow cross-links between xylan chains and between xylans and lignin. Such cross-linkages have a negative effect on maize cell walls degradability (Grabber et al., 1998, 2009; de Oliveira et al., 2015). Downregulation of *BAHD* genes encoding acyltransferases with FAT activity in Brachypodium, rice and Setaria results in a drop in GAX-bound ferulates (Piston et al., 2010; Buanafina et al., 2016; de Souza et al., 2018). Expression analysis in maize hinted five genes encoding BAHD acyltransferases as potential *FAT* genes, however no functional analysis has been undertaken yet to validate their function (Chateigner-Boutin et al., 2016). The *seedling ferulate ester* (*sfe)* maize mutant was selected from a transposon mutant stock, by screening for seedlings with reduced ferulate ester content, but the mutated gene causing this phenotype is not yet identified (**Table 1**) (Jung and Phillips, 2010; Hatfield et al., 2018). The *sfe* mutant biomass does not only show reduced ferulate levels, but also has lower lignin levels, and improved rumen digestibility as evidenced by its performance in animal feeding trials (Jung et al., 2011).

### Agronomic performance

Concerns often arise that plants with reduced lignin levels would have increased susceptibility to pests, diseases, reduced biomass yield and lodging. Indeed, lignin does play an important role in these agronomic traits and some maize mutants with altered lignin were reported to have such unfavorable characteristics (**Table 1**) (Oliveira et al., 2020; Ren et al., 2020). On the other hand, it seems that lignin content variations within the natural maize population have little discernible impact on its agronomic performances (Pedersen et al., 2005; Barrière, 2017; Manga-Robles et al., 2021). In addition, promising inbred lines have already been developed with enhanced fermentable sugar yields that rival or surpass the *bm* mutant. These lines exhibit superior tolerance to fall armyworm compared to their isogenic counterparts (Vermerris et al., 2007). Similarly, another study also observed no close correlation between insect susceptibility and the lignin content (Williams et al., 1998).

The *bm3* mutants have demonstrated the most significant improvement in *in vitro* cell wall digestibility as compared to other *bm* mutants, making them the most promising among all *bm* mutants for breeding purposes. However, a *bm3* mutation often results in a reduction of 15 to 20% in dry matter yield as compared to their non-mutant isogenic controls (Inoue and Kasuga, 1989). In contrast, two studies reported no biomass yield reductions associated with the *bm3* mutation in certain genetic backgrounds as compared to their isogenic control lines (Weller et al., 1985; Gentinetta et al., 1990). The *bm3* mutants are also often associated with reduced stalk strength, increased susceptibility towards pathogens and drought, and precocious senescence (Nicholson et al., 1976; Zuber et al., 1977; Vermerris et al., 2010; Akhter et al., 2018). However, several studies were not able to detect a correlation between increased lodging susceptibility and *bm3* mutants (Weller et al., 1985; Inoue and Kasuga, 1989). Recently, some of these pleiotropic effects of a *bm3* mutation, such as precocious senescence and low drought tolerance, were counteracted by the introgression of a *BRACHYTIC2* (*BR2*) mutation, resulting in a *bm3 br2* double mutant (Landoni et al., 2022). In conclusion, there are strong interactions between the gene of choice, the genetic background and the environment in which the plants are grown. Therefore, the agronomic performance will largely be determined by these factors (Pedersen et al., 2005).

## Conclusion and future perspectives

Lignin plays a fundamental role in various agronomically relevant traits. Lignin offers tolerance against biotic stresses, provides structural support to stems to prevent lodging, yet it also acts as a biomass recalcitrance factor limiting cell wall degradability. Current evidence suggests that reducing lignin content in maize can be achieved without compromising plant growth and biomass yield by choosing the right gene or by introgression of the corresponding mutation into the most appropriate genetic background. There is still considerable potential for new lignin-engineering strategies in maize, as many genes involved in lignin biosynthesis and its regulation have yet to be investigated (Barrière et al., 2015; Penning et al., 2019). Genomic studies have shown that lignin biosynthesis genes are often part of multigene families, of which in most cases only a single member has been analyzed. Knocking-out multiple members of the same gene family, which has only now become possible by CRISPR-based gene editing, is a promising strategy to further improve the processability of lignocellulosic biomass. In addition, CRISPR-based gene editing allows the generation of a range of alleles, not only knock-out alleles, but also weak alleles that should allow the tuning of lignin content and composition without affecting yield, as illustrated by editing *CCR2* in poplar (De Meester et al., 2020). Furthermore, by analyzing lignin mutants and transgenics, it has become clear that lignin is tolerant towards large compositional shifts. This opens perspectives to redirect the lignin pathway towards the overproduction of rare natural monomers, or even to let the plant synthesize alternative monomers by expressing exotic genes. For example, ectopic overexpression of the two penultimate genes of the scopoletin biosynthesis pathway in lignifying cells in Arabidopsis has resulted in the incorporation of scopoletin into the lignin polymer, making it more susceptible to alkaline pretreatments (Hoengenaert et al., 2022). Several other examples and strategies exist and have been reviewed (Mottiar et al., 2016; Vanholme et al., 2019; de Vries et al., 2021).

For future research and applications, a few critical factors need to be considered with regard to the genotype to work with, and the environment where the experiments are carried out. First, it needs to be recognized that most studies on the effects of lignin engineering on digestibility have been conducted in greenhouses. The main reason for this is that the effect of a genetic modification is easier to determine in a stable greenhouse environment. Experiments with transgenic plants under field conditions require a regulatory permit, which discourages most researchers from conducting field trials. Nevertheless, field trials are essential to validate the effect of the mutation. Field trials represent a realistic environment with fluctuating weather conditions (wind, UV radiation, rain, drought), exposure to abiotic stressors and variations in soil type and soil microorganisms. Furthermore, the planting density in the field differs from that of plants grown in pots under the controlled greenhouse settings. Thus, results obtained in the greenhouse may not always align with observations made under field conditions (Tuberosa, 2012; Nelissen et al., 2014). When improving a commercially relevant trait, it is therefore advisable to conduct the experiments directly in the field to select the most promising mutants, and only then study their more subtle effects in a greenhouse setting for purely scientific purposes. Second, it is important to recognize that genotypes typically used in experiments and transformation (laboratory strains) are not optimized for cell wall degradability. As a result, genomic modifications can lead to substantial improvements in the digestibility in these genotypes, whereas the improvement does not necessarily translate into elite inbreds or their hybrids. It will be essential to either edit elite inbred lines if transformation protocols exist for them, or to use other methods such as transgenerational editing. Importantly, when the mutations are recessive, both elite inbred lines will need to be edited in order to create a mutant hybrid. Only when the mutant hybrid outperforms, and preferentially in different environments, the strategy can be considered successful. Navigating the future of maize breeding requires embracing cutting-edge gene editing technology. By addressing the challenges associated with genotype selection and field validation, we can adopt a sharper approach to develop modern maize varieties that contribute to a sustainable agriculture and bio-based economy.

## Author contributions

YV: Writing – original draft, Writing – review & editing. ADM: Writing – original draft, Writing – review & editing. HM: Writing – original draft, Writing – review & editing. RV: Writing – original draft, Writing – review & editing. WB: Writing – original draft, Writing – review & editing.

## Footnotes

1 Given the land required for growing animal feed, methane emissions from ruminants and animal welfare, the ecological and ethical implications of consuming meat and dairy are a rising matter of concern. Despite this, such practices remain widespread (Dixon et al., 2023; Henchion and Zimmermann, 2021).
